# Improving health literacy of patients with pulmonary embolism through evidence-based health information: a feasibility study

**DOI:** 10.3389/fpubh.2026.1814447

**Published:** 2026-05-13

**Authors:** Simone Fischer, Julia Reizner, Anja Kalch, Helena Bilandzic, Thomas M. Berghaus, Christine Meisinger, Inge Kirchberger

**Affiliations:** 1 Epidemiology, Faculty of Medicine, University of Augsburg, Augsburg, Germany; 2 Department of Media, Knowledge and Communication, University of Augsburg, Augsburg, Germany; 3 Department of Cardiology, Respiratory Medicine and Intensive Care, University Hospital Augsburg, Augsburg, Germany; 4 Medical Faculty, Ludwig Maximilians University of Munich, Munich, Germany

**Keywords:** evidence-based patient information, feasibility, health literacy, health promotion, pulmonary embolism

## Abstract

**Background:**

Pulmonary embolism (PE) is a common condition with significant physical and psychological impairments. This study was intended to examine the feasibility of how evidence-based health information on PE in the form of a brochure can be distributed and used to strengthen PE-specific health literacy.

**Materials and methods:**

Patients with PE were recruited during their hospital stay and were randomized in an intervention group which was provided with the brochure and a control group. Questionnaires were completed at baseline and four months after PE. Primary focus of the study were feasibility metrics such as recruitment and dropout rates, suitability of randomization processes, and the acceptance of the intervention. Health literacy was assessed using the Health Literacy Pulmonary Embolism (HeLP) questionnaire to gather information about expected effect sizes of the intervention. Further patient-oriented and information-related outcomes (e.g., health-related quality of life, mental well-being, communication with treating physicians and relatives) were assessed as secondary outcomes during follow-up. Additional qualitative interviews in the intervention group were conducted for further information on acceptance and usage patterns of the brochure.

**Results:**

At baseline, 116 of 249 hospitalized patients with PE were enrolled in the study. Seventy-five participants (intervention: 33, control: 42) completed the follow-up, representing a response rate of 65%. PE-specific health literacy was significantly improved in the domain ‘dealing with PE-related health information’ in the intervention group (3.86 vs. 3.43, *p* = 0.030, r = 0.30). Participants of the intervention group showed also better PE-related knowledge compared to the control group (45% vs. 17% with ≥ 2 correct items, *p* = 0.007, Cramer’s V = 0.29) and a stronger feeling of being informed (6.00 vs. 4.14, *p* < 0.001, r = 0.50). Qualitative interviews underlined predominant usage of the brochure during post-acute phase. Patients rated the patient information as easily accessible and as a trustworthy source and highlighted the emotional support through narratives of other patients.

**Conclusion:**

Provision of evidence-based health information via a brochure is feasible and may enhance PE-specific health literacy and patient knowledge. Challenges regarding distribution strategies, effective implementation and long-term effects should be considered in future studies.

## 1 Introduction

Pulmonary embolism (PE) is potentially life-threatening, the third most common cardiovascular disease and a major cause of hospitalization and morbidity (1, 2). After PE, more than half of patients suffer from persistent symptoms such as shortness of breath and reduced physical performance and 18% from right heart failure (2, 5). Mental health may also be impaired after PE with qualitative studies describing the diagnosis as traumatic and reporting concerns about side effects of anticoagulant medication. Patients report symptoms such as hypervigilance, rumination, sleep disturbances, loss of energy and feelings such as frustration, uncertainty and panic (3, 4, 6, 7). Between 17 and 24% of patients showed symptoms of depression or anxiety up to two years after PE (8). Coping with PE requires both general health literacy (e.g., understanding information about the disease) and disease-specific health literacy (e.g., dealing with anticoagulant medication). Low health literacy is associated with several negative health outcomes, poorer disease management in chronic conditions, lower medication adherence, higher hospitalization and rehospitalization rates, and increased morbidity and premature death (9–12). Improving health literacy may therefore enhance health outcomes. In a systematic review of health literacy interventions, an improvement in health literacy was achieved in 15 of 22 studies (13). Patients with PE could also benefit from interventions to cover information deficits, to improve health literacy and find an active and independent way of dealing with the disease and its consequences. An evidence-based health information brochure for patients with PE was developed in a previous research project (14). While electronic or mobile health information and literacy is a fast growing field, it has been shown that older people still prefer to use written information in brochures or books (15, 16). Another aspect is the inclusion of narratives in the form of personal experiences by patients with similar conditions. Including such narratives in health information can change beliefs, attitudes, and intentions, especially regarding increasing healthy behaviors (17). However, it remains unclear whether providing such written information is associated with an improvement of the health literacy of patients with PE compared to patients lacking this information. Large scale implementation requires evidence of effectiveness, ideally from a multicenter randomized controlled trial (RCT). Given the substantial resources required for an RCT, a feasibility study will help to clarify in advance when and how the brochure should be distributed, how it will be accepted and used, and what additional strategies can be employed to encourage the best possible use of health information. This study aims to inform the feasibility of a RCT that examines whether evidence-based health information in the form of a brochure can improve PE-specific health literacy, as well as other patient-oriented and information-related endpoints (e.g., health-related quality of life, mental well-being, communication with treating physicians and relatives). Therefore, this study serves as preliminary work for a subsequent larger multicenter RCT, focusing on feasibility metrics such as recruitment and randomization processes, retention rates, acceptance of the intervention and questionnaires, and expected effect sizes of the intervention in relation to different outcomes.

## 2 Materials and methods

### 2.1 Study design

**Figure 1:**
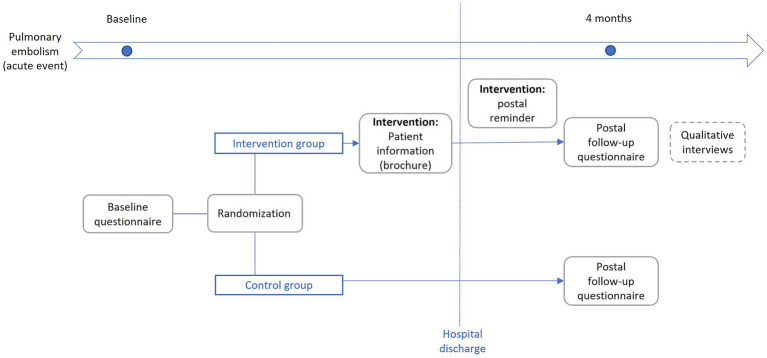
Study design.

The feasibility study was planned as a randomized controlled two-arm, (parallel-group) interventional trial in a single university hospital. Adult patients (age ≥ 18 years) hospitalized with incident or recurrent confirmed PE diagnosis based on multidetector CT pulmonary angiography or ventilation–perfusion lung scanning in the University Hospital Augsburg between May 2024 and April 2025 were assessed for eligibility. Exclusion criteria were severe cognitive impairment (e.g., dementia), severe visual impairment or alexia, insufficient understanding of the German language and life expectancy of less than one year. During the hospital stay, the intervention group received written evidence-based information on PE in the form of a brochure. The control group did not receive the brochure. Both groups received a postal follow-up questionnaire 4 months after enrollment. We assumed 4 months to be an appropriate time period, because studies have shown that commonly patients show an improvement of physical health and coping with the acute PE event during the first months (18, 19). On the other hand, the “post PE syndrome” is defined as cardiac, pulmonary, or functional limitations that persist 3 months after the acute event (20). Furthermore, we intended to avoid patients being lost to follow-up due to early in-patient rehabilitation. If participants did not respond, a postal reminder was sent first, followed by an attempt to contact them by telephone. Additionally, a small selection of the intervention group was invited to qualitative interviews in person after they responded to the postal follow-up. The detailed study design is described in Figure 1. The study protocol was approved by Ethics Committee of the Ludwig-Maximilians-Universität München. Participants from the control group also received the brochure after the postal follow-up was completed. Based on our previous experience with studies involving patients with PE at the same hospital, we expected to recruit an average of 10 participants per month. Therefore, we aimed to recruit a total of approximately 120 participants (60 per group) over a 12-month period. This number also aligns with recommendations for sample size in feasibility studies (21, 22). Successful retention was defined as participants returning the follow-up questionnaire. A response rate of at least 60% was expected to inform feasibility of the study design.

### 2.2 Recruitment and randomization

Participants were recruited during hospitalization shortly after their PE. A trained study nurse provided detailed information about the study, obtained the written consent and collected baseline data. Participants were randomly allocated to one of the two study arms using simple block randomization to ensure balanced group sizes over time in a 1:1 ratio. A statistician, who was not part of the project team, predetermined the block sizes and generated the random allocation sequence. The allocation was printed on paper and placed in an opaque envelope by a data manager of the institute who also stored the envelopes for the duration of the study. All envelopes were sequentially numbered and prepared prior to start of the recruitment process. Allocation was concealed from the recruiting study nurse until completion of consent and baseline data collection and was then conducted by opening the previously sealed envelope at the patient’s bedside.

### 2.3 Intervention

The intervention comprised the provision of evidence-based information on PE in the form of a brochure which was newly developed as a part of a previous large research project involving different professional disciplines (psychologist, health scientist, communication scientists, epidemiologists, internists/pulmonologists, health insurance representatives), and patients with PE (14). It includes information on pathophysiology, risk factors, treatment, progression, and prevention of PE, as well as narratives from patients about their experiences with life after PE. The information is written in easily understandable language, and the brochure has a visually appealing design to support understanding and reading flow. Additionally, a reminder was sent to the participants of the intervention group two months after study enrolment. The reminder was a small postal flyer to support the use of the brochure.

### 2.4 Outcomes and measures

At baseline, socio-demographic data, comorbidities and subjective health status [EQ5D5L (23, 24)] were assessed.

In the follow-up questionnaire, PE-specific health literacy was assessed using the Health Literacy Pulmonary Embolism (HeLP) questionnaire. The questionnaire was developed with comprehensive involvement of patients with PE in a previous research project and contains 23 Items in four domains: dealing with PE-related health information, disease management, health-related selfcare, and social support (25).

Further secondary outcomes were PE-specific quality of life (PEmb-QoL, total score and 6 subdomains: frequency of PE-related complaints, limitations in activities of daily living, work-related problems, social limitations, intensity of complaints, and emotional complaints) (26), health-related self-efficacy (SES6G) (27), depression and anxiety (HADS score) (28), quality of the Physician-Patient Relationship (PRA-D, subdomain communication) (29), use of health care services [based on FIMA (30)], feeling of being informed (self-developed items, e.g.: “How well informed do you feel about the effects of pulmonary embolism on everyday life (e.g., reduced performance)?”), PE-related knowledge (self-developed items, e.g.: “Which statement about the bleeding risk when taking anticoagulants is incorrect?”). The intervention group completed additional questions on the use of the brochure, subsequent communication, and about support received through the information in the brochure (emotional, informational, interactional), e.g., “The brochure encouraged me in dealing with pulmonary embolism.”, “The brochure provides me with additional information that I was not previously familiar with.”, or “The brochure made conversations with the physician easier” (Supplementary Table S1).

As part of feasibility assessment, detailed reasons for exclusion during the recruitment process, dropout rates and their reasons, completeness of questionnaires and preferences regarding the brochure use were collected.

### 2.5 Statistical analysis

Descriptive statistics were stratified by study arm. As a part of feasibility, missing data patterns were examined overall and specifically for the single questionnaires. Comparisons between intervention and control group at baseline and four months were performed using Student’s t-test or Mann–Whitney U-test for continuous variables and chi-square or Fisher’s exact test for categorical variables. Rank-biserial correlation and Cramer’s V were calculated as effect sizes. An alpha level of 0.05 was used for hypothesis testing. Since these are preliminary investigations from a feasibility study with explorative character, no adjustment for multiple testing was performed. Complete case analyses were performed due to the low number of missing values. Number of cases included is provided for each test separately. All analyses were conducted with the statistic software R version 4.4.2.

### 2.6 Qualitative interviews

In addition to the questionnaires, we aimed to gather further information on how the brochure was used and perceived by conducting qualitative interviews with a small subsample of the intervention group. All participants of the intervention group were approached by a postal invitation after completion of the follow-up questionnaire. Two weeks later, they were contacted by a researcher to arrange the interview. Prior to the face-to-face interview, they received postal information about the study and had to sign additional written consent for participating in the interviews. Interviewees were invited until theoretical saturation for feasibility was achieved.

The interviews were semi-structured and focused on the timing of using the brochure, perceived usefulness for the patients themselves and in communication with physicians and relatives. All interviews were audio-recorded, transcribed verbatim, and analyzed using qualitative content analysis following Kuckartz’s approach (31). Coding was performed independently by two researchers with discrepancies resolved through discussion. Themes were iteratively refined until consensus was reached.

## 3 Results

### 3.1 Participant characteristics

**Figure 2:**
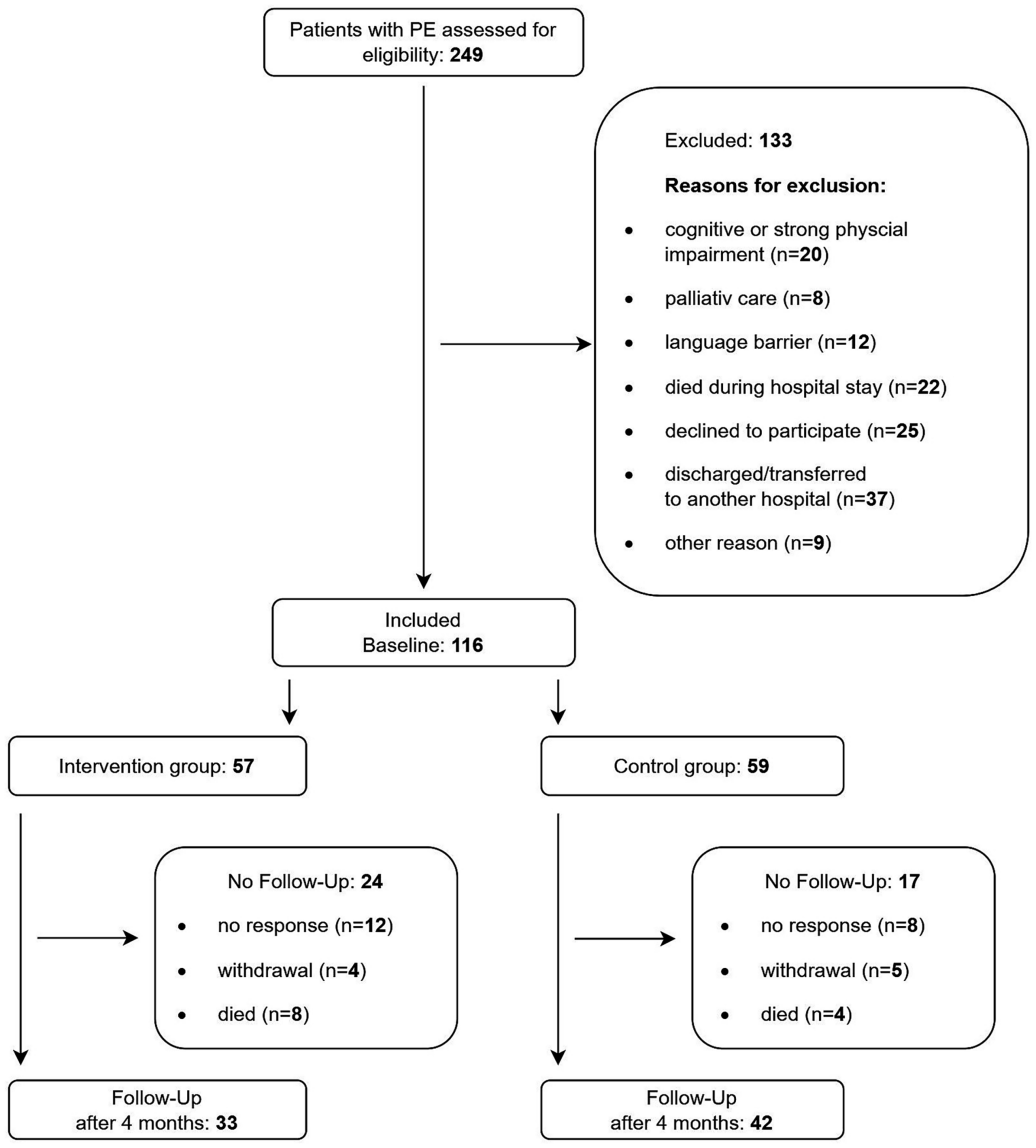
Flow chart of recruitment and follow-up process. Intervention group: brochure with PE-specific health information. Control group: usual care after PE. PE = pulmonary embolism.

Over a 10-month period, 249 patients with PE were assessed for eligibility. Of these, 133 patients were excluded for several reasons (Figure 2), with early discharge or transfer to another hospital being the most common. Other reasons for exclusion included cognitive impairment or language barriers. In total, 116 patients (53% female) participated in the study, with 57 randomly assigned to the intervention group and 59 to the control group. The mean age was 66 (± 14.7) years, and 11% had previous PE. Baseline characteristics did not differ significantly between intervention and control group (Table 1).

**Table 1:** Patient characteristics stratified by study arm.

Characteristic	*N*	Intervention group *N* = 57^1^	Control group *N* = 59^1^	*p*-value^2^
Age	116	63.7 (15.0)	67.4 (14.3)	0.183
Gender (female)	116	31 (54.4)	31 (52.5)	0.842
Living alone	113	22 (40.0)	16 (27.6)	0.163
Native language (German)	115	52 (92.9)	55 (93.2)	>0.999
School education (≥ 9 years)	112	32 (59.3)	30 (51.7)	0.423
Employed	112	24 (43.6)	19 (33.3)	0.262
History of pulmonary embolism	116	8 (14.0)	5 (8.5)	0.343
EQ VAS	116	50.0 (40.0, 65.0)	50.0 (40.0, 75.0)	0.523
EQ-5D-5L index	113	0.6 (0.2, 0.8)	0.7 (0.5, 0.9)	0.080
Thrombophilia	110	9 (17.0)	10 (17.5)	0.938
Diabetes Type 2	112	8 (14.8)	13 (22.4)	0.303
Hypertension	112	23 (42.6)	31 (53.4)	0.251
Heart failure	110	10 (18.2)	9 (16.4)	0.801
Myocardial infarction	111	3 (5.6)	5 (8.8)	0.717
Stroke	114	3 (5.5)	5 (8.5)	0.718
Anxiety	112	7 (13.0)	10 (17.2)	0.528
Depression	114	10 (17.9)	12 (20.7)	0.702
Other psychiatric disease	111	5 (9.3)	2 (3.5)	0.263
Pulmonary hypertension	111	0 (0.0)	2 (3.4)	0.496
Cancer	113	19 (33.9)	19 (33.3)	0.947

^1^Mean (SD); Median (Q_25_, Q_75_); *n* (%).

^2^Student’s t test; Mann-Whitney U-test; Chi-squared test; Fisher's exact test.

Seven persons from the intervention group participated in the qualitative interviews. Recruitment was stopped after seven interviews, as we considered the subsample sufficiently diverse to represent different perspectives, and no new themes emerged. Participants were 29 to 70 years old, three were female, six of them were still taking anticoagulant medication. Time between the interview and their PE event were six to seven months. All characteristics of the interviewees are presented in Supplementary Table S2.

### 3.2 Acceptance and response

Response rate for the follow-up questionnaire was 65%. In the intervention group 42% and in the control group 29% of the participants were lost to follow-up (Figure 2). Baseline characteristics of the intervention and the control group who completed the follow-up survey did not differ (Supplementary Table S3). Patients who dropped out of the study differed from those who completed the follow-up in several aspects: they were more likely to live alone (46% vs. 27%, *p* = 0.041), had lower levels of school education (39% vs. 64% of ≥ 9 years, *p* = 0.009), were less frequently currently employed (26% vs. 45%, *p* = 0.043), and more often had diabetes (30% vs. 13%, *p* = 0.023) (Supplementary Table S4).

Missing values in the follow-up questionnaire made up 2.8% of the data in general for items used in both study arms. Outcome-specific missing values did not exceed 3.7% for one questionnaire. (Supplementary Table S5). Missing values of items that focused on the use of patient information and were only included in the questionnaire for the intervention group made up 2.2%.

Given the small sample size, the following presented group differences should be seen as exploratory rather than proof of effectiveness of the intervention.

### 3.3 PE-specific health literacy

**Figure 3:**
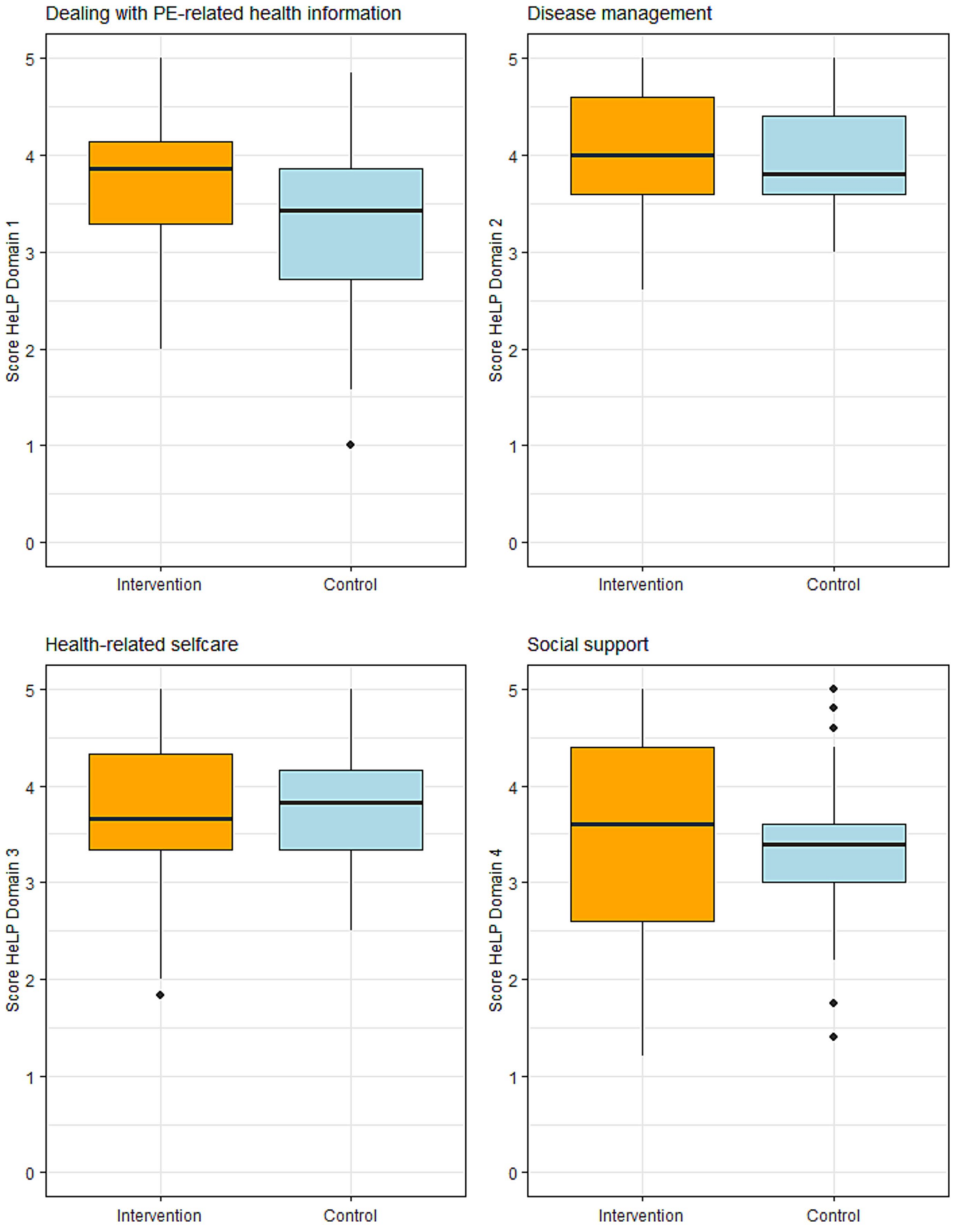
PE-specific health literacy in the four HeLP domains (dealing with PE-related health information, disease management, health-related self-care, social support) 4 months after the acute PE in the intervention group (orange, *n* = 33) and in the control group (blue, *n* = 41). Higher scores indicate better health literacy. PE = pulmonary embolism. HeLP = Health Literacy Pulmonary Embolism.

The intervention group showed statistically significant better scores in the HeLP domain ‘dealing with PE-related health information’ (Medians: 3.86 vs. 3.43, *p* = 0.030) with a medium effect size of 0.30. The scores in the domains ‘Disease management’ and ‘Social support’ were also slightly higher in the intervention group compared to the control group but were lacking statistical significance (Table 2; Figure 3).

**Table 2:** Differences in outcomes between intervention and control group after 4 months.

Outcome	N	Intervention group *N* = 33^1^	Control group *N* = 42^1^	*p*-value^2^	Effect size^3^
Dealing with PE-related health information (HeLP)	74	3.86 (3.29, 4.14)	3.43 (2.71, 3.86)	0.030	0.30
Disease management (HeLP)	74	4.00 (3.60, 4.60)	3.80 (3.60, 4.40)	0.686	0.06
Health-related selfcare (HeLP)	74	3.67 (3.33, 4.33)	3.83 (3.33, 4.17)	0.600	0.07
Social support (HeLP)	74	3.60 (2.60, 4.40)	3.40 (3.00, 3.60)	0.563	0.08
EQ-5D-5L index	75	0.85 (0.67, 1.00)	0.82 (0.66, 0.92)	0.373	0.12
EQ VAS	75	70.0 (50, 85.0)	68.0 (50.0, 80.0)	0.720	0.05
PE-related knowledge (≥2 correct items)	75	15 (45)	7 (17)	0.007	0.29
Feeling of being informed	73	6.00 (4.93, 6.79)	4.14 (3.00, 5.71)	<0.001	0.50
Depression (HADS)	72	4.0 (1.0, 10.0)	4.0 (2.0, 8.0)	0.806	0.03
Anxiety (HADS)	75	6.0 (2.0, 9.0)	4.0 (2.0, 8.0)	0.496	0.09
HADS Score	72	12.0 (4.0, 18.0)	10.0 (5.0, 15.0)	0.561	0.08
Health-related self-efficacy	73	7.33 (5.50, 9.17)	7.67 (6.50, 9.00)	0.933	0.01
Physician-Patient Relationship (PRA-D, subdomain communication)	75	24.0 (17.0, 25.0)	22.0 (19.0, 25.0)	0.715	0.05
Hospitalization in the last 4 months	74	12 (38)	23 (55)	0.141	0.13
Outpatient treatment in the last 4 months	73	11 (35)	16 (38)	0.819	0.00
Number of visits to physicians (≥4) in the last 4 months	75	14 (42)	22 (52)	0.392	0.00
PEmb-QoL Total Score	71	33.0 (8.0, 53.0)	41.0 (10.0, 56.0)	0.518	0.09
PEmb-QoL Frequency of complaints	75	16.0 (3.0, 25.0)	14.0 (3.0, 25.0)	0.735	0.05
PEmb-QoL Limitations in activities of daily living	72	30.0 (12.0, 58.0)	44 (12.0, 67.0)	0.458	0.10
PEmb-QoL Work-related problems	74	75 (0.0, 100.0)	100 (0.0,100.0)	0.477	0.09
PEmb-QoL Social limitations	74	25.0 (0.0, 50.0)	50.0 (0.0, 50.0)	0.333	0.13
PEmb-QoL Intensity of complaints	74	30.0 (10.0, 40.0)	30.0 (10.0, 50.0)	0.519	0.09
PEmb-QoL Emotional complaints	74	28.0 (8.0, 42.0)	22.0 (12.0, 44.0)	0.957	0.00

^1^Median (Q_25_, Q_75_); *n* (%).

^2^Mann-Whitney U-test; Pearson's Chi-squared test; Fisher's exact test.

^3^Rank-biserial correlation or Cramer’s V.

### 3.4 Secondary outcomes

The intervention group had significantly better PE-related knowledge (higher proportion (≥2) of correct responses to six knowledge-related items; 45% vs. 17%, *p* = 0.007) and higher scores in the questions about the feeling of being informed (Medians: 6.00 vs. 4.14, *p* < 0.001) with medium to large effect sizes of 0.29 and 0.50, respectively (Table 2).

Subjective health status (EQ5D index and EQ VAS) were slightly higher in the intervention group and PE-related quality of life (PEmb-QoL) showed a tendency toward better scores for the intervention group in the total score and in the domains ‘limitation in activities of daily living’, ‘work-related problems’, and ‘social limitations’ but did not yield statistical significance. The number of uses of health care services (i.e., visits to physicians, inpatient, and outpatient treatment in the hospital in the last four months) were higher in the control group. In addition, the scores of the subdomain ‘communication’ as part of the physician-patient relationship were slightly higher in the intervention group compared to the control group. In contrast, the total HADS score, the HADS anxiety score and the subdomain ‘emotional complaints’ of the PEmb-QoL were slightly worse in the intervention group but also without showing statistically significant differences (Table 2).

### 3.5 Use of patient information brochure

Most participants preferred the printed patient information (64%) and used it one to three times during the last four months (76%). The majority (76%) first read the brochure directly after the PE during the hospital stay and 53% reported this as the point in time at which the patient information is most helpful.

Participants rated the chapters about causes of the PE, symptoms/acute phase, and treatment as the most important chapters of the brochure (Supplementary Figure S1).

On a scale from 1 (=not at all interesting/relevant) to 7 (=very interesting/relevant), participants rated the relevance of the information with a mean of 5.72 (±1.44) and the interest with a mean of 5.88 (±1.41). On the scale from 1 (= ‘The information was completely new to me’) to 7 (= ‘I was already familiar with this information’) the participants rated on average with 2.78 (±1.96).

Participants responded on a scale of 1 (= very unlikely) to 7 (= very likely) with a mean of 3.72 (± 2.26) that they would talk about the brochure with relatives or friends, or their physicians (mean = 3.42, SD = ± 2.14), respectively. The chance that they would search for further information regarding PE (e.g., training plans or opportunities for exchange about their disease), was rated with a mean of 4.40 (± 2.29).

Thirty-four percent of the participants reported that relatives have read the brochure, 13% recommended it to relatives or friends, and 19% recommended their physician to read the brochure.

Support that was received through the information in the brochure was rated on a scale from 1 (=‘I do not agree at all’) to 7 (=‘I totally agree’), with a mean of 4.24 (±1.92) for emotional support, 5.55 (±1.25) for informational support, and 4.26 (±2.18) for interactional support (facilitating conversations with physicians and relatives about PE).

### 3.6 Qualitative interviews

Three key informational themes of the brochure emerged from the interviewees: cause of PE, risk of recurrence, and patient narratives.

#### 3.6.1 Patient narratives

Many participants valued the inclusion of reports of patients’ experience because they conveyed the feeling of not being alone and allowed comparison with other affected individuals.

*“And reading it all over and over again and saying, yes, okay, she’s about the same age as me, or she’s still in pain, or that’s how it went for her, actually helped me a little more than just reading about what the disease means.”* (female, 29 years).

*“It helped me a little bit, in the sense that I realized, okay, what happened to me has happened to others too, […] I feel more confident, it’ll be okay.”* (male, 43 years).

At the same time, several participants reported the identification with these narratives to be difficult because their personal trajectories did not match the narratives presented in the brochure.

Preferences for the type of informational content varied. Some participants stated that objective facts about the disease were more important than patient narratives, whereas others judged the narratives to be more helpful than facts.

#### 3.6.2 Impact on patients

Overall, the patient information evoked predominantly positive psychosocial effects. Interviewees described feelings of relief and reassurance and reported that the material provided support and a sense of safety during recovery.

*“For me, it was reassuring, let’s say. Okay, you can train and you just stop when you can’t go on anymore, but you’re not damaging your lungs or anything like that, so it really helped in everyday life.”* (male, 43 years)

*“Because you feel like, okay, I don’t have to fight my way through the jungle of information now, someone else has done that for me, and that’s fine.”* (female, 29 years)

Some also reported a feeling of being informed and a stronger awareness about their condition after reading the brochure. Conversely, some information or narratives were perceived as extreme or strongly negative, so that they remained in their mind.

*“That didn’t really help me, because I’m the type of person who never remembers numbers, but if they’re bad, then I do. And in that case, it really was enough, that 5 or 10%, to tell me, yes, you’ll get another one because you’re unlucky. […] It didn’t really take away my fear, it just gave my fear a number.”* (female, 29 years).

#### 3.6.3 Accessibility and trust

The content of the brochure was judged mainly comprehensible, and the graphical layout was found to be helpful for the reading flow. Overall, trustworthiness of the information included was rated as high. Several participants considered it equally credible to physicians’ statements, and many rated it more trustworthy than information found on the internet. Notably, explicit evidence labeling (i.e., statements about the evidence level and the related references) were not noticed by most of participants. However, some reported that an active engagement with the evidence labeling would influence their appraisal of the information.

*“I have a tendency to take ‘strong evidence’ more seriously than ‘weak evidence’.”* (male, 43 years).

#### 3.6.4 Use patterns

Many participants read the brochure in its entirety, while others selectively searched for personally relevant topics. There was a clear tendency toward the use in the first weeks after the event. Regarding timing, some participants reported that the acute phase represented a period of high information need and available time to read, whereas others found themselves unable to engage with the brochure due to physical or emotional constraints right after the PE.

*“I only leafed through it a little in the hospital, but I was totally tired and could not really take it in.”* (female, 49 years).

Generally, opinions were mixed about the optimal timing. Some participants did not recommend distribution during the acute phase because of information overload, while others favored early provision during hospitalization.

#### 3.6.5 Communication

Participants described the brochure as helpful for relatives and as a tool for preparing consultations with physicians, although several interviewees reported that they had not discussed the brochure with anyone.

*“When it came to scheduling [for rehabilitation], I went through the whole thing again, made a few notes, what’s important to me, what I wanted to address […] because the brochure also brought up questions that I wanted to ask the doctor.”* (male, 64 years).

Overall, the brochure was perceived as supportive information in the time after the pulmonary embolism. Regarding distribution, some participants recommended combining the provision of the brochure with a conversation with a healthcare professional. An attending physician or nurse may help to filter the information that applies to oneself.

*“But simply having someone sitting next to you who goes through this with you […], so that you can find yourself in the brochure.”* (male, 43 years).

## 4 Discussion

Overall, this study aimed to generate preliminary results of feasibility processes and metrics, as well as exploratory group differences regarding the provision of evidence-based health information for patients with PE.

The main aspects of feasibility were recruitment, response and design of the intervention (e.g., timing). Recruitment of patients with PE in the clinical context was challenging because a substantial proportion of eligible patients were discharged early or encountered only in the emergency department, which limited the opportunities for personal approach and study enrolment. Language barriers accounted for approximately 9% of exclusions, underscoring the need for translation and cultural adaptation of patient information in this setting to ensure equal access and to reduce systematic exclusion of non-native speakers.

Dropout rates during follow-up were high. Patients lost to follow-up tended to have lower levels of education and were more likely to live alone – characteristics of a group that should be prioritized when providing accessible patient information. The intervention arm showed a higher dropout rate than the control arm, possibly due to the longer questionnaire, which may reflect increased respondent burden. Another explanation could be that their need for support in coping with the illness was satisfied, or that their recovery process was already quite advanced. These aspects may have reduced their motivation to complete an additional questionnaire in that phase. The participants of the control group, on the contrary, were still waiting to receive the patient information, which may have served as an extrinsic motivator. Despite the high dropout rates, the returned questionnaires had a low percentage of missing items, indicating that the instruments were acceptable to the respondents. However, for a future RCT, strategies to improve retention must be implemented. Those should include more frequent or earlier reminders for the follow-up survey, tightening the follow-up questionnaire, and explicitly educating participants about the importance of the follow-up survey during recruitment. Furthermore, telephone assistance with completing the survey could be offered to increase retention in the group of participants living alone or showing lower levels of education.

Participants rated topics that predominantly referred to the acute phase of PE as most important. Usage patterns indicated that the brochure was most frequently read during the acute phase and the first weeks thereafter. These findings suggest that the immediate post-event period represents the most useful time window for distributing the brochure. This time frame is in line with a conceptual model of informational needs for decision making of patients with acute myocardial infarction. According to Decker et al., informational needs increase after the acute event and treatment have passed, and patients then wish to take on a more active role. The need for information increases particularly in the period after discharge, when direct access to healthcare providers is limited (32).

Apart from feasibility, we also exploratorily investigated potential effects of the patient information. Statistically significant differences in the domain ‘dealing with PE-related health information’, in items regarding PE-specific knowledge and in the items about feeling of being informed suggest that the brochure may be able to address informational needs and convey core information in an accessible format. No improvements were detected in the other domains of PE-specific health literacy. Contrarily, the development study of the HeLP questionnaire found improvements across all four domains after receiving the brochure, likely due to the greater statistical power in that study (25).

Reading the brochure did not substantially encourage communication about PE with relatives or physicians, as shown by both the quantitative questionnaire data and the qualitative interviews. This may indicate that the brochure sufficiently met patients’ immediate informational needs, reducing their perceived need for additional discussion.

Qualitative data, and to some extent the questionnaire data, indicated that the brochure may induce anxiety in some cases, particularly when patients read it while still overwhelmed in the acute phase and thus have limited capacity to process information. In such situations, involvement of a supportive third person (e.g., a nurse, physician, family member or a self-help group) may be helpful to interpret and contextualize the content. The latter was also a suggestion mentioned in the qualitative interviews. However, implementing this support into routine care might pose a challenge. Conversely, without access to evidence-based information, many patients seek information online, which has been associated with increased health anxiety (33, 34). Carefully developed, disease-specific health information, created with input from health care professionals and patients, may help mitigate this effect.

A larger sample will be required to confirm our exploratory results. Effects on further outcomes, and potential mediation and moderation effects should also be examined in future studies. Different psychological, cognitive or social factors could play a role in a more complex causal chain of linking information provision to health literacy, to general health outcomes, and behavioral and psychological outcomes. In previous studies health literacy was associated with self-care activities including indirect pathways via self-efficacy, empowerment, social support, and health care communication (35). While a review reported predominantly positive associations of health literacy and behavioral outcomes, studies found inconsistent associations between health literacy and emotional status (36). This complex interplay must be considered when investigating health literacy interventions. Studies focusing on distribution strategies of patient information and the integration into the care process to improve long-term effects on disease management propose repeated activation through contact with health care providers or text messages (37, 38). These strategies and their potential for improving the care for patients with PE especially during the transition between inpatient and outpatient settings could also be tested in a larger study. Since providing patient information can be considered a cost-effective intervention, the inclusion of an economic evaluation is of interest and may provide important information for the transfer of such intervention to other diseases.

### 4.1 Strengths and limitations

The strengths of this study are the randomized controlled trial design, the collection of a variety of patient-reported outcome data and additional qualitative data from personal interviews. The brochure which served as the intervention in this study was carefully developed in a large previous research project involving multiple professions and strong patient involvement.

However, one limitation is the small sample size, but the aim of the study was to conduct a preliminary investigation of group differences and to establish feasibility metrics. A postal reminder sent to the intervention group to encourage use of the brochure may be seen as another limitation since it could have affected the usage pattern of the brochure. The challenge during recruitment related to early discharges may have led to a selection bias and limits generalizability to all patients with PE. The differences in dropout rates and reasons for dropping out between the intervention and control group may have also led to an attrition bias undermining the comparison between the two groups. Recruitment from one single university hospital also limits generalizability of the findings about recruitment and follow-up processes to other settings. The processes may be applicable for other large university hospitals but not for smaller specialized treatment centers or district hospitals with fewer cases of PE.

### 4.2 Practice implications

The findings support the brochure’s role in addressing informational needs in the post-acute phase. Future dissemination strategies should consider ways to reach patients who are discharged quickly or immediately after their stay at the emergency department. Strategies could include electronic delivery, early distribution by caregivers in the hospital or provision by general practitioners. The timing of the distribution of the brochure should be optimized for the early post-acute period, and complementary approaches that facilitate processing of information and enhance comprehension to reduce potential anxiety should be evaluated during implementation.

## 5 Conclusion

This feasibility study demonstrates acceptability of the patient information about PE and provides preliminary indications about potential effects on informational outcomes. Practical challenges such as early discharge of patients with PE, high dropout rates, and language barriers, should be considered in subsequent RCTs and implementation studies. These feasibility metrics provide important information for several future studies.

## Data Availability

The raw data supporting the conclusions of this article will be made available by the authors, without undue reservation.
