# Supplementary material for: Improving health literacy of patients with pulmonary embolism through evidence-based health information: a feasibility study
Source: Front Public Health. 2026 May 13;14:1814447. doi: 10.3389/fpubh.2026.1814447 (PMC13212078; doi:10.3389/fpubh.2026.1814447)
Supplement: Supplementary file 1 [file Data_Sheet_1.pdf]

Supplementary Material

Improving health literacy of patients with pulmonary embolism through evidence-based health information: a feasibility study

Simone Fischer[1]*, Julia Reizner[1], Anja Kalch[2], Helena Bilandzic[2], Thomas M. Berghaus[3,4], Christine Meisinger[1], Inge Kirchberger[1]

[1] Epidemiology, Faculty of Medicine, University of Augsburg, Augsburg, Germany

[2] Department of Media, Knowledge and Communication, University of Augsburg, Augsburg, Germany

[3] Department of Cardiology, Respiratory Medicine and Intensive Care, University Hospital Augsburg, Augsburg, Germany

[4] Medical Faculty, Ludwig Maximilians University of Munich, Munich, Germany

Table S1: Measures by time point and study arm

**Measure**	**Baseline**	**Follow up Intervention group**	**Follow up Control group**
Socio-demographic data	x		
Comorbidities	x		
Subjective health status (EQ-5D-5L)	x	x	x
PE-specific health literacy (HeLP)		x	x
PE-specific quality of life (PEmb-QoL)		x	x
Depression and anxiety (HADS score)		x	x
Self-efficacy (SES6G)		x	x
Quality of the Physician-Patient Relationship (PRA-D, subdomain communication)		x	x
Use of health care services (based on parts of (FIMA)		x	x
PE-related knowledge; feeling of being informed		x	x
Use of the brochure (timing, frequency, etc.)		x	
Subsequent communication (regarding brochure)		x	
Support (emotional, informational, interactional)		x	

Table S2: Sample characteristics of interviewees in the intervention group

**Nr.**	**Age**	**Gender**	**Number of PE events**	**Time between interview and last PE event**	**Family status**	**School education**	**Ongoing anticoagulative medication**
1	70	female	1	7 months	widowed	10 years	yes
2	29	female	1	6 months	married	≥ 12 years	yes
3	43	male	1	7 months	single	≥ 12 years	no
4	64	male	1	7 months	married	10 years	yes
5	42	male	4	6 months	married	≥ 12 years	yes
6	43	male	1	6 months	married	≥ 12 years	yes
7	49	female	3	7 months	married	≥ 12 years	yes

Table S3: Patient characteristics by study arm (with follow-up completed, n=75)

**Characteristic**	**N**	**Intervention group** N = 33[1]	**Control group** N = 42[1]	**p-value**[2]
Age	75	62.9 (15.0)	64.7 (15.0)	0.605
Gender (female)	75	18 (54.5%)	20 (47.6%)	0.551
Living alone	74	12 (37.5%)	8 (19.0%)	0.077
Native language (German)	74	30 (93.8%)	40 (95.2%)	>0.999
School education (≥ 9 years)	73	22 (68.8%)	25 (61.0%)	0.491
Employed	73	15 (48.4%)	18 (42.9%)	0.639
History of PE	75	4(12.1%)	2 (4.8%)	0.395
EQ VAS	75	50.0 (45.0, 70.0)	52.5 (45.0, 75.0)	0.893
EQ-5D-5L index	72	0.7 (0.3, 0.8)	0.8 (0.5, 0.9)	0.134
Thrombophilia	71	5 (16.7%)	6 (14.6%)	>0.999
Diabetes Type 2	72	4 (12.9%)	5 (12.2%)	>0.999
Hypertension	73	13 (41.9%)	22 (52.4%)	0.377
Heart failure	71	5 (15.6%)	4 (10.3%)	0.722
Myocardial infarction	73	1 (3.1%)	3 (7.3%)	0.626
Stroke	74	0 (0.0%)	4 (9.5%)	0.129
Anxiety	73	5 (15.6%)	7(17.1%)	0.868
Depression	73	5 (15.6%)	8 (19.5%)	0.667
Other psychiatric disease	72	3 (9.4%)	2 (5.0%)	0.650
Pulmonary hypertension	72	0	0	-
Cancer	72	9 (28.1%)	15 (37.5%)	0.402

[1]Mean (SD), Median (Q_{25}, Q_{75}); n (%)

[2] Student's t test; Mann Whitney U-test; Pearson's Chi-squared test; Fisher's exact test

Table S4: Characteristics of participants who were lost to follow up compared to participants with follow-up

		Follow up completed		
**Characteristic**	**N**	**No**<br>N = 41[1]	**Yes**<br>N = 75[1]	**p-value**[2]
Age	116	68.7 (14.0)	63.9 (15.0)	0.087
Gender (female)	116	24 (58.5%)	38 (50.7%)	0.417
Living alone	113	18 (46.2%)	20 (27.0%)	0.041
Native language (German)	115	37 (90.2%)	70 (94.6%)	-
School education (≥ 9 years)	112	15 (38.5%)	47 (64.4%)	0.009
Employed	112	10 (25.6%)	33 (45.2%)	0.043
History of PE	116	7 (17.1%)	6 (8.0%)	0.216
EQ VAS	116	50.0 (30.0, 60.0)	50.0 (45.0, 70.0)	0.068
EQ-5D-5L index	113	0.6 (0.2, 0.8)	0.7 (0.5, 0.9)	0.098
Thrombophilia	110	8 (20.5%)	11 (15.5%)	0.505
Diabetes Type 2	112	12 (30.0%)	9 (12.5%)	0.023
Hypertension	112	19 (48.7%)	35 (47.9%)	0.938
Heart failure	110	10 (25.6%)	9 (12.7%)	0.085
Myocardial infarction	111	4 (10.5%)	4 (5.5%)	0.442
Stroke	114	4 (10.0%)	4 (5.4%)	0.448
Anxiety	112	5 (12.8%)	12 (16.4%)	0.611
Depression	114	9 (22.0%)	13 (17.8%)	0.591
Other psychiatric disease	111	2 (5.1%)	5 (6.9%)	>0.999
Pulmonary hypertension	111	2 (5.1%)	0 (0.0%)	0.121
Cancer	113	14 (34.1%)	24 (33.3%)	0.930

[1]Mean (SD); Median (Q_{25}, Q_{75}); n (%)

[2] Student's t test; Mann-Withney U-test; Pearson's Chi-squared test; Fisher's exact test

Table S5: Missing data in follow-up questionnaires (n=75)

**Questionnaire**	**Missing fields (in %)**
Disease specific quality of life (PEmb-QoL)	3.6
Health Literacy Pulmonary Embolism (HeLP)	3.4
Subjective health status (EQ5D5L and EQ-VAS)	0
Depression anxiety (HADS)	0
Health-related self-efficacy (SES6G)	2.9
Quality of the Physician-Patient Relationship (PRA-D, subdomain communication)	2.7
Use of health care services (FIMA)	3.7
Feeling of being informed and PE-related knowledge	3.1

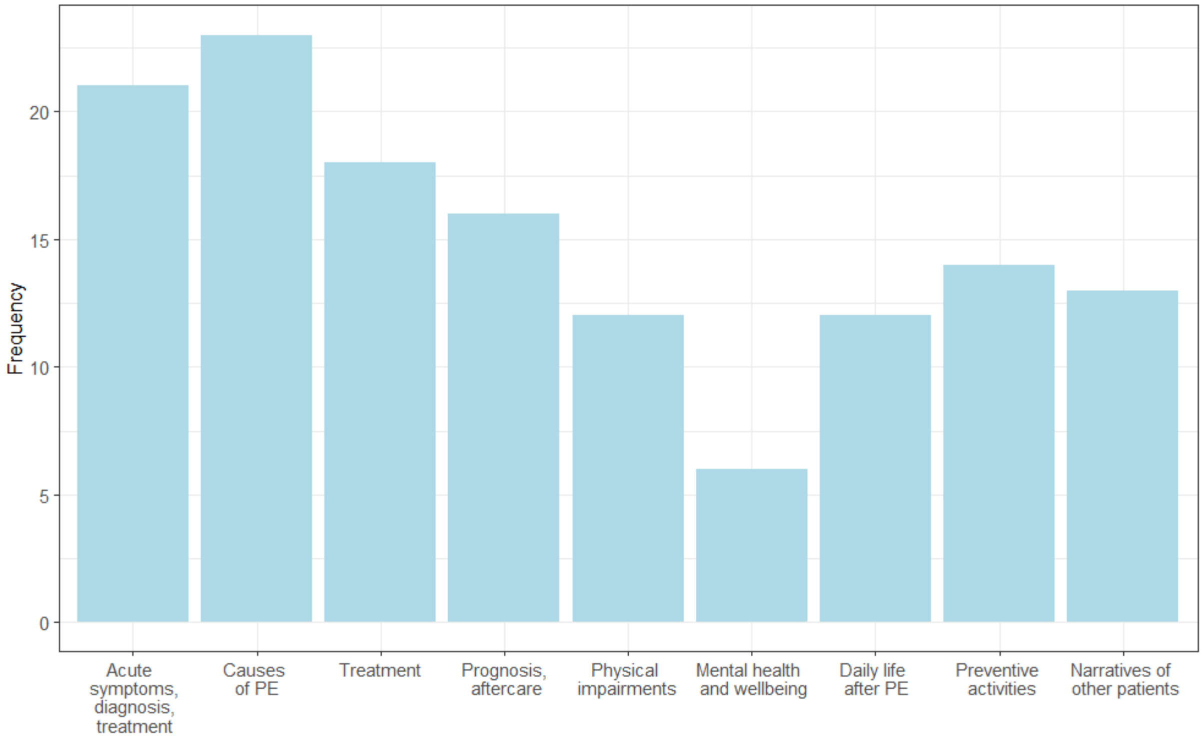

Figure S1: Responses of the intervention group to the question about which chapter in the brochure was particularly relevant and helpful. Multiple entries were possible.
